# Genetic Alteration Analysis of IDH1, IDH2, CDKN2A, MYB and MYBL1 in Pediatric Low-Grade Gliomas

**DOI:** 10.3389/fsurg.2022.880048

**Published:** 2022-04-28

**Authors:** Orit Barinfeld, Alon Zahavi, Shirel Weiss, Helen Toledano, Shalom Michowiz, Nitza Goldenberg-Cohen

**Affiliations:** ^1^ Sackler Faculty of Medicine, Tel Aviv University, Tel Aviv, Israel; ^2^ The Krieger Eye Research Laboratory, Bruce and Ruth Rappaport Faculty of Medicine, Technion-Israel Institute of Technology, Haifa, Israel; ^3^ Ophthalmology Department, Rabin Medical Center – Beilinson Hospital, Petach Tikva, Israel; ^4^ Department of Pediatric Oncology, Schneider Children’s Medical Center of Israel, Petach Tikva, Israel; ^5^ Department of Neurosurgery, Schneider Children’s Medical Center of Israel, Petach Tikva, Israel; ^6^ Department of Ophthalmology, Bnai-Zion Medical Center of Israel, Haifa, Israel

**Keywords:** glioma, pediatric, molecular, IDH1, MYB, MYBL1, CDKN2A

## Abstract

**Objective:**

To investigate pediatric low-grade gliomas for alterations in *IDH1, IDH2, CDKN2A, MYB, and MYBL1*.

**Materials and Methods:**

DNA and RNA were extracted from 62 pediatric gliomas. Molecular methods included PCR, RT-PCR, and RNA sequencing; Sanger sequencing was used for validation.

**Results:**

Analysis for hotspot genetic alterations in *IDH1* R132 and *IDH2* R172 (45 and 33 samples) was negative in all cases. *CDKN2A* deletions were detected in exons 1 and 2 in 1 (pleomorphic xanthoastrocytoma) sample of 9 samples analyzed. Of 10 samples analyzed for *MYB* translocation, 4 each were positive for translocations with exon 2 and exon 3 of *PCDHGA1*. Six samples showed *MYBL* rearrangement. The lack of *IDH1/2* genetic alterations is in accordance with the literature in pediatric tumors. Alterations in *MYB, MYBL* were recently reported to characterize diffuse grade II, but not grade I, gliomas.

**Conclusion:**

We optimized methods for analyzing gene variations and correlated the findings to pathological grade. The high incidence of MYB and MYBL need further evaluation. We also compared DNA, RNA, and RNA sequencing results for fusion, translocation, and genetic alterations. More accurate identification of the underlying biology of pediatric gliomas has implications for the development of targeted treatment.

## Introduction

Brain tumors are the most common solid tumors in children, with a prevalence of 2.4–4 per 100,000. More than 40% are low-grade gliomas (LGGs) originating from glial cells, usually astrocytes (1–5). LGGs comprise a heterogeneous group of tumors. The most common is pilocytic astrocytoma which accounts for 30% of all central nervous system tumors in children (6).

LGGs (grade I-II) have less aggressive biological behavior than higher-grade gliomas (grade III-IV), with better response to treatment and better prognosis. The first line of treatment is surgical resection unless the tumor is located in less accessible areas such as the diencephalon, brain stem, or optic pathways (2, 6) when chemotherapy or radiation may be necessary. All these modalities especially radiation have significant side effects, such as cognitive impairment, vascular injury, and secondary malignancies. Pilocytic astrocytoma is associated with a 10-year overall survival rate of more than 85% (3), but the rate is much lower for grade II tumors (4, 6).

Until fairly recently, relatively little was known about the molecular changes that promote the formation or growth of LGGs in children. There is now clear evidence that dysregulated signaling of the Ras/Raf/MEK/ERK mitogen-activated protein kinase (MAPK) pathway plays an important role in glioma biology (5). Serine/threonine-protein kinase B-raf is a member of the Raf family of kinases and serves as a downstream effector in the MAPK pathway. Molecular studies have reported ERK/MAPK activation via focal gains at band 7q34 resulting in a fusion gene, *BRAF-KIAA1549,* in up to 80% of pilocytic astrocytomas of the cerebellum (7, 8). Activation of the Ras/Raf pathway can also result from oncogenic mutations of the B*RAF* gene itself, usually substitution of glutamic acid for valine at amino acid 600 (V600E), although they are less commonly identified in pediatric LGG (3%–10%) (6, 7, 9). These findings highlight the importance of the ERK/MAPK pathway both in the development of pediatric LGG and as a possible therapeutic target. Studies in various phases of development are already in progress assessing both BRAF and downstream MEK inhibitors for the treatment of gliomas in children (6, 10–12). Gene fusions may also provide a means of molecular classification of pilocytic astrocytomas in the future.

The commonest alterations in LGG in children are in BRAF. Either a fusion or a genetic alteration. In our previous study of *BRAF* genetic alterations in 45 samples of pediatric LGGs (13), we found an increased *BRAF* copy number in 18 of the 47 primary samples tested; 15 of them (83.3%) were pilocytic astrocytomas. A *BRAF* genetic alteration was found in 3 of 48 primary tumors, all with a normal *BRAF* copy number and no *GNAQ* genetic alteration. One sample had a *GNAQ209* genetic alteration (Q209P626) with a normal *BRAF* gene. None of the tumors had a *GNA11Q209* genetic alteration. Recurrent or progressive tumors, analyzed in 4 patients, had the same molecular genotype as their primary. The upregulation of the RAS/MAPK pathway is a major factor in the molecular pathways of pediatric LGGs and data are accumulating from large studies (6).

Aberrations in *PTEN* (14) and *IDH1/2* (15) genes have previously been detected in adult high-grade astrocytomas (15, 16) but not in any pediatric astrocytoma samples examined. This supports previous findings that some molecular changes in astrocytomas may be age-dependent. CDKN2A genetic alterations are found in low grade gliomas often with BRAF genetic alterations and correlate with later clinical progression (6, 15).

Recently, attention has been addressed to three new genes that may be involved in the genetic profile of grade II diffuse gliomas: *MYB, MYBL1,* and *FGFR1*. *MYB*, an oncogene not previously implicated in gliomagenesis, was found to be activated in a diverse subset of pediatric LGGs (17). Novel rearrangements were identified, usually translocation or fusion with exon 2 or exon 3 of *PCDHGA1.* Rearrangements were also identified in the *MYBL1* oncogene in addition to duplication and fusion of the tyrosine kinase domain in *FGFR1* (6). These events may be exclusive to grade II gliomas and are likely to have an important role in tumorigenesis (18). The findings have implications for improving the identification and treatment of grade II gliomas, especially those involving the thalamus that are not amenable to complete surgical resection.

The aim of the present study was to screen pediatric grade I and II LGGs for genetic alterations in candidate genes *IDH1/2, CDKN2A, MYB and MYBL1* in 62 pLGG in order to investigate their potential involvement in pediatric gliomagenesis. Molecular methods included PCR, RT-PCR, and RNA sequencing; Sanger sequencing was used for validation. Nowadays, next generation sequencing (NGS) panel for wide screening may be recommended as an alternative.

## Results

### Low-Grade Gliomas

Paraffin-embedded or fresh-frozen samples were collected from 62 pediatric patients with various grade I (*n* = 45) or grade II (*n* = 17 LGGs, as follows: pilocytic astrocytoma (*n* = 34), ganglioglioma (*n* = 8), diffuse astrocytoma (*n* = 6), pleomorphic xanthoastrocytoma (*n* = 6), pilomyxoid astrocytoma (*n* = 3), subependymal giant cell astrocytoma (*n* = 2), astroblastoma (*n* = 1), gangliocytoma (*n* = 1), oligodendroglioma (*n* = 1). Data on patient demographics, tumor location, treatment, response to treatment, and survival are shown in Table 1.

**Table 1:** Patient data and analysis for IDH1/2 in gliomas grade I and grade II.

Pt.	Age at 1st operation	M/F	Diag-nosis	Grade	Location	2nd surgery	NF-1	Chemo-therapy	Radio-therapy	Death	IDH2 mutation	IDH1 mutation
1	0.9	F	PA	1	OTG	N	N	Y	N	N	N/A	N/A
2	16.9	F	PA	1	Chia – ht	Y	N	Y	N	N	N/A	N/A
3	23.4	F	PA	1	OTG	N	N	Y	N	N	N/A	N/A
4	2	M	FA	2	Chia – ht	Y	N	Y	Y	N	N/A	N/A
5	3	M	PA	1	Chia – ht	Y	N	Y	N	Y	N/A	neg
6	1.1	M	PA	1	Chia – ht	N	N	Y	N	N	neg	neg
7	8.4	M	PA	1	Chia – ht	N	N	N	N	N	neg	neg
8	4.6	F	PA	1	OTG	N	Y	Y	N	N	neg	neg
9	3.8	F	PA	1	Chia – ht	N	N	N	N	N	neg	neg
10	1.4	F	PA	1	Chia – ht	N	N	Y	N	N	neg	neg
11	2.7	M	PA	1	PF	Y	N	Y	N	N	N/A	N/A
12	10.11	M	PA	1	Temporal	N	Y	N	N	N	neg	N/A
13	16.6	F	PA	1	PF	N	N	N	N	N	neg	N/A
14	16.1	M	PA	1	PF	Y	N	N	N	N	N/A	neg
15	4.1	M	PA	1	PF	N	N	N	N	N	N/A	N/A
16	12.7	F	PA	1	Temporal	Y	N	N	N	N	N/A	neg
17	14.11	M	PA	1	Pineal	N	N	N	N	N	N/A	N/A
18	5	F	PA	1	PF	N	N	N	N	N	neg	neg
19	8.1	F	PA	1	PF	N	N	N	N	N	N/A	N/A
20	2	F	PA	1	PF	Y	N	N	N	N	neg	neg
21	14.1	M	PA	1	PF	Y	N	N	N	N	neg	neg
22	6.1	F	PA	1	PF	Y	N	Y	N	N	neg	neg
23	12.4	M	PA	1	PF	N	N	N	N	N	neg	neg
24	1.9	M	PA	1	PF	Y	N	N	N	N	neg	neg
25	6.8	F	PA	1	Ventricle	N	N	N	N	N	N/A	neg
26	15	M	PA	1	PF	N	N	N	N	N	neg	neg
27	2.5	M	Astroblastoma	2	Temporal	Y	N	N	N	N	neg	neg
28	14.3	F	PA	1	Temporal	N	N	N	N	N	neg	neg
29	22	F	FA	2	Temporal	N	N	N	Y	N	neg	neg
30	7	F	PA	1	Ventricle	Y	N	N	N	N	N/A	N/A
31	11	F	PA	1	Brainstem	N	N	Y	N	N	N/A	neg
32	2.2	M	Gangliocytoma	1?	PF	N	N	N	N	N	neg	neg
33	0.1	F	FA	2	Temporal	N	N	N	N	N	neg	neg
34	4.2	M	PA	1	Thalamus	N	N	N	N	N	neg	neg
35	10.1	F	PA	1	PF	N	Y	Y	N	N	neg	neg
36	3.5	M	PMA	1	PF	N	N	N	N	N	neg	neg
37	17	F	Ganglioglioma	1	Brainstem	Y	N	N	N	N	N/A	N/A
38	3.1	M	Ganglioglioma	1	Temporal	N	N	N	N	N	neg	neg
39	12.8	M	Ganglioglioma	1	Temporal	N	N	N	N	N	N/A	neg
40	14.4	M	Ganglioglioma	1	Occipital	Y	N	N	N	N	N/A	N/A
41	14	F	Ganglioglioma	1	Temporal	Y	N	N	N	N	N/A	neg
42	14.2	F	Ganglioglioma	1	Temporal	N	N	N	N	N	N/A	N/A
43	9.11	M	Ganglioglioma	1	Temporal	N	N	N	N	N	neg	neg
44	12.11	F	PXA	2	Temporal	Y	N	Y	Y	Y	neg	neg
45	11	F	PXA	2	Temporal	Y	N	N	N	N	neg	neg
46	14.9	F	PXA	2	Temporal	Y	N	N	N	N	neg	neg
47	5.8	M	SEGA	?1	Ventricle	N	N	N	N	N	neg	neg
48	3.4	M	SEGA	?1	Ventricle	Y	N	N	N	N	neg	N/A
49	1	F	FA grade II	2	temporal	N	N	Y	N	N	N/P	neg
50	4.5	M	GG	1	temporal	N	N	Y	N	N	N/P	neg
51	14.5	F	FA grade II	2	intraventricular	N	N	Y	N	N	N/P	neg
52	18.1	M	FA grade II	2	Bithalamic	N	N	Y	N	N	N/P	neg
53	3.6	F	PMA grade II	2	PF	N	N	Y	N	N	N/P	neg
54	12	F	PXA grade II	2	intraventricular	N	N	Y	N	N	N/P	neg
55	1.1	F	PMA grade II	2	hypothalamic	N	N	Y	N	N	Neg	Neg
56	3.5	M	PA	1	PF	N	N	N	N	N	Neg	neg
57	1.5	F	PA	1	Optic nerve	N	N	N	N	N	Neg	neg
58	9	F	PA	1	PF	N	N	N	N	N	Neg	neg
59	6.1	F	PA	1	PF	N	N	N	N	N	Neg	neg
60	17.3	F	PXA	?1	intraventricular	N	N	Y	N	N	N/P	Neg
61	N/A	M	PXA Grade II	2	Temporal lobe, brain stem	N	N	Y	Y	Y	N/A	N/A
62	N/A	M	Oligodendroglioma Grade II	2	Left frontal lobe	N	N	?	?	?	N/A	N/A

*PA, pilocytic astrocytoma (grade I); FA, diffuse astrocytoma; PMA, Pilomyxoid astrocytoma (grade I); PXA, pleomorphic xanthoastrocytoma (grade II); SEGA, subependymal giant cell astrocytoma (grade II); OTG, optic tract glioma; chia-ht, chiasmatic – hypothalamus; PF, posterior fossa; NF-1, neurofibromatosis type 1; N/P, not performed; Neg, negative.*

### IDH1 and IDH2 Analysis by PCR DNA

Polymerase chain reaction (PCR) DNA analysis for *IDH1* and *IDH2* was performed in 45 and 34 samples, respectively. All findings were negative. Of the 62 samples, 14 did not show a sequence for either gene (N/A) and 7 were evaluated for only one gene (N/P) (Table 1).

### CDKN2A Analysis by RT-PCR

**Figure 1:**
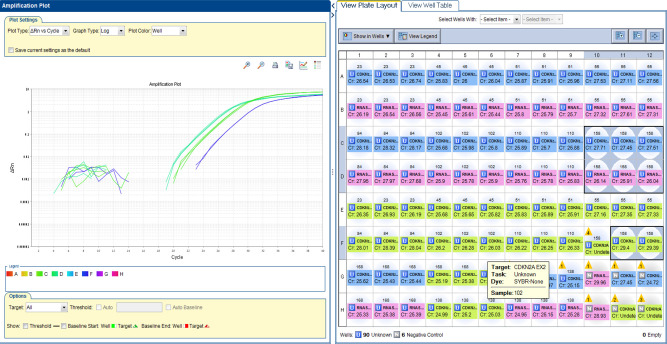
CDKN2A plot. A deletion was detected in exon 1 and exon 2 of CDKN2A in one sample (no. 54) of pleomorphic xanthoastrocytoma. RTPCR plot, showing Reference gene RNaseP - 26 Ct. Please note CDKN2A Exon 1–27 Ct (middle green) and CDKN2A Exon 2–29 Ct (right blue). A ratio of <0.5 was considered a deletion.

Real-time PCR (RT-PCR) was performed on 9 grade II pediatric glioma samples. A deletion was detected in exon 1 and exon 2 of *CDKN2A* in one sample (no. 54) of pleomorphic xanthoastrocytoma. The cycle threshold (Ct) values are shown in **Figure 1**.

### MYB Translocation Analysis by RACE

**Figure 2:**
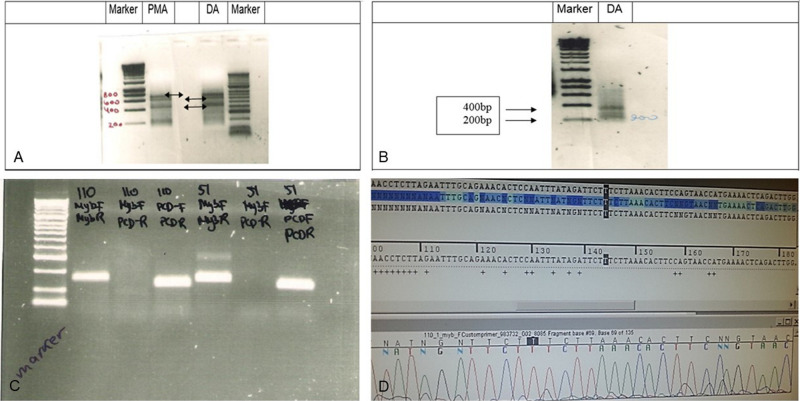
RACE analysis for MYB translocation. (**A**) RACE analysis of Grade II glioma samples 55 (PMA,102) and 51 (DA). RACE gel results showing the bands (amplicons by primers combination 1 + 3). Note the bands at 700k, 600k and 450k (arrows) in both samples If translocation occurred, then the PCR reaction will give a shorter amplicon. (**B**) RACE results primers 2 + 3 in sample 51 show 2 bands, 200 and 380 bp. (**C**) PCR products in gel. Left to right: Marker, Sample 53 (110) primer combination of MYB F + R, MYB F+ PCDHGA1 R, PCDHGA1 F + R, sample 51 combination of primers of MYB F + R, MYB F+ PCDHGA1 R, PCDHGA1 F + R. Only 53(110) showed light band in MYB F+ PCDHGA1 R combination, suggesting translocation of the MYB. (**D**) *S*equence of the normal MYB in sample 55. Validation PCR only showed the normal MYB sequence.

Two samples of grade II LGG were initially analyzed for *MYB* translocation using rapid amplification of cDNA ends (RACE): one diffuse astrocytoma (no. 51) and one pilomyxoid astrocytoma (no. 55) (**Figure 2A–D**). When primers 1 + 3 were used, the RACE gel showed bands at 700, 600, and 450 k in both samples. With the primer combination 2 + 3, two bands were detected, at 200 and 380 bp.

### MYB Analysis by RACE, Sanger Validation

Although we detected RACE products of translocation in the gel, direct sequencing showed only the normal *MYB* sequence, probably because the bands were weak (**Figure 2D**). The PCR results with a combination of 3 primers included MYB (forward + reverse)[(primers (1) + (2)], MYB (forward) + PCDHGA1(reverse)[primers(1) + (4)], PCDHGA1(forward + reverse) [primers (3) + (4)] in samples 53 and 51. A light band was detected in sample 53 using the 1 + 4 [MYB (forward) + PCDHGA1(reverse)] primer combination.

### MYB Translocation Analysis by RT-PCR

**Figure 3:**
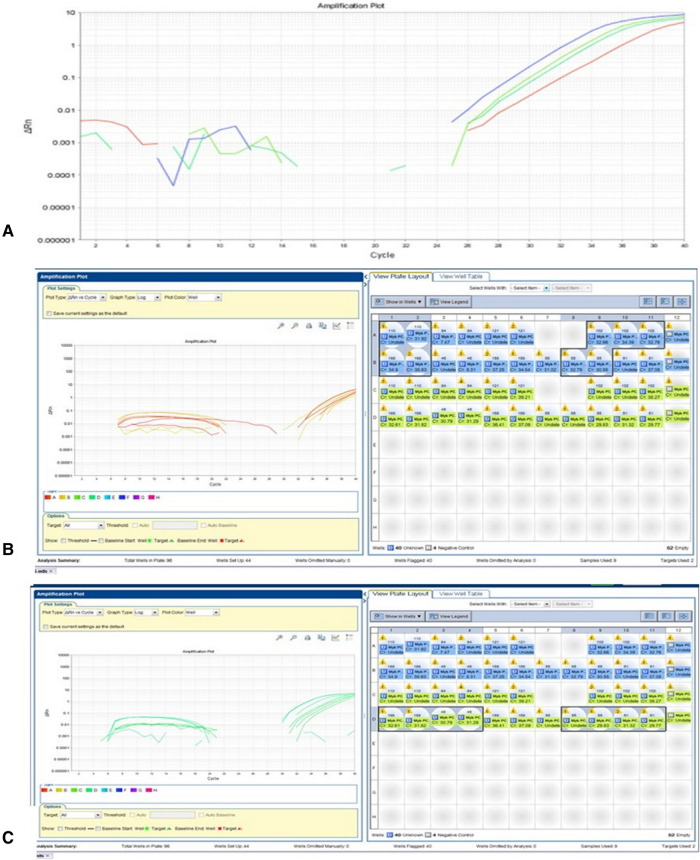
MYB translocation with exon 2 or 3 of PCDHGA1 By RT-PCR. (**A**) As predicted, the product was detected in sample 53 (110) with primers MYB F + R (blue) and PCDHGA1 F + R (upper green); sample 51 showed product in primers MYB F + R combination (lower green) and PCDHGA1 F + R (red). (**B**) RT-PCR results of MYB exon 9 and exon 2 of PCDHGA1. Note 4 positive samples for this translocation 53(110), 55(102) – PMA; 168- oligodendroglioma; 60(55)-PXA. (**C**) RT-PCR results of MYB exon 9 and exon 3 of PCDHGA1. MYB forward & PCDHGA1 reveres show product only in translocation samples. Note 4 positive samples for this translocation (51- DA; 50(45)- ganglioglioma;168 - oligodendroglioma; 60(55) - PXA).

RT-PCR with a different combination of primers was used as an alternative method to detect *MYB* translocation with *PCDHGA1* in 10 samples (Table 2). In samples without *MYB* translocation, the reactions with primers 1 + 2 or 3 + 4 led to a product, and in samples with *MYB* translocation with exon 2 of *PCDHGA1*, the reactions with primers 1 + 4 led to a product. Product was detected in sample 53 with primers 1 + 2 and 3 + 4 and also in sample 51 (**Figure 3A–C**). Five samples were positive for translocation of exon 9 of *MYB* with exon 2 of *PCDHGA1*: nos. 53, 55 (pilomyxoid astrocytoma), no. 62 (oligodendroglioma), and no. 60 (pleomorphic xanthoastrocytoma), and 4 were positive for translocation of exon 9 of *MYB* with exon 3 of *PCDHGA1* (primer combination 5 + 6): nos. 51 (diffuse astrocytoma), 50 (ganglioglioma), 62 (oligodendroglioma), and 60 (pleomorphic xanthoastrocytoma).

**Table 2:** List of primers for RT-PCR reaction for MYB expression.

Symbol	Chr	Orientation	Location	Primer
1	6	sense	MYB-Exon 9	AGCAAGGTGCATGATCGTC
2	6	Anti-sense	MYB-Exon 9	GGGGGTGGAAGTTAAAGAAGG
3	5	sense	PCDHGA1 Exon2	CAGCACCCCAGTCTTTACTTG
4	5	Anti-sense	PCDHGA1 Exon2	CGGTGTCATCGCCATTTT
5		sense	MYB –Exon9	GAACCACACATGCAGCTACC
6		Anti- sense	PCDHGA1 Exon3	TGCAGCATCTCTGTGTCAAA

### Comparative MYB and BRAF Analysis by RT-PCR

Of the 10 samples, 5 were positive for translocation of *MYB* with exon 2 of *PCDHGA1*, and 4 were positive for *MYB* translocation with exon 3 of *PCDHGA1*. All samples positive for a translocation were negative for *BRAF* except sample 51.

### MYBL1 Rearrangement by PCR and RT-PCR (DNA Level)

To determine whether *MYBL1* duplication-truncation was present in grade II pediatric LGGs, PCR for genomic DNA and RT-PCR were used. Ten samples of grade II gliomas were analyzed at the DNA level **(Tables 3 and 4**, **Figure 3**). A band at 350 bp on the gel represents a PCR product of the reaction. Eight samples were positive, as follows: no. 50 (ganglioglioma), 52, 51 and 49 (diffuse astrocytoma), 55 and 53 (pilomyxoid astrocytoma), 54 (pleomorphic xanthoastrocytoma), and 62 (oligodendroglioma). The remaining sample, no. 60 (pleomorphic xanthastrocytoma), was negative.

**Table 3:** BRAF and MYB gene rearrangement.

Patient number	Diagnosis	Location	Sex	BRAF V600E	MYB- PCDHGA1 Exon 2	MYB - PCDHGA1 Exon 3	MYBL1 DNA
49	FA grade 2	Temporal	F	Negative	neg	neg	+
50	GG grade 2	Temporal lobe	M	Negative	neg	+	+
51	FA grade 2	Intraventricular	F	Positive	+*	+	+
52	FA grade 2	Bithalamic	M	Negative	neg	neg	+
53	PMA grade 2	PF	F	Negative	+*	neg	+
54	PXA grade 2	intraventricular	F	positive	neg	neg	neg
55	PMA grade 2	Hypothalamic	F	Negative	+	neg	+
60	PXA grade 2	Intraventricular	F	No data	+	+	neg
61	PXA grade 2	Temporal lobe/ Brain stem	M	Positive	neg	neg	No data
62	Oligodendroglioma grade 2	Lt frontal	M	Negative	+	+	neg

**Table 4:** Results of MYBL-1 analysis in DNA and cDNA of same samples.

Patient number	DNA reslts for MYBL-1 by real time PCR	Gel of DNA for MYBL-1	cDNA results MYBL-1 by real time PCR
49	Positive	Positive	Negative
50	Positive	Positive	Positive
51	Positive	positive	Negative
52	Positive	Positive	Positive
53	Positive	Positive	Negative
54	Positive	Positive/questionable	Negative
55	Positive	positive	Negative
60	Negative	negative	Positive
61	No data	No data	Negative
62	Positive	negative	Negative

When PCR products of genomic DNA were run on gel**,** we detected a band at 350 bp in 6 samples: nos. 49, 51 and 52 (diffuse astrocytoma), 50 (ganglioglioma, and 55 and 53 (pilomyxoid astrocytoma). In one sample (no. 60, pleomorphic xanthoastrocytoma), the band was undetected, and in one (no. 54, pleomorphic xanthoastrocytoma), the results were equivocal. Sample 62 (oligodendroglioma) was negative in the gel but positive on RT-PCR. The data are summarized in Table 4.

It was clear that there were discordant results for *MYBL1*, so we appllied several different methods in an attempt to reach conclusive results.

### Analysis of MYBL1 by RT-PCR (cDNA Level)

**Figure 4:**
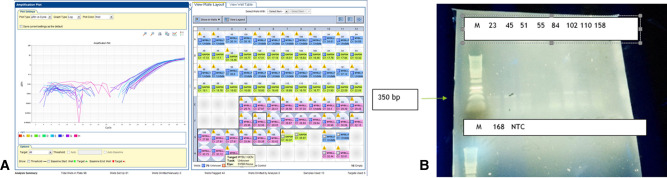
MYBL-1 duplication–truncation by RT-PCR. Genomic DNA of Glioma grade II: Negative (*n* = 1): 60(55)-PXA. Positive (*n* = 8): 49–55 and 168. Key of gel order: 50(45) GG, 52(84) FA, 51 FA, 49(23) FA, 55(102) PMA, 53(110) PMA, 54(158) PXA, 168 ODG. Of note, sample 168 was negative on gel, although positive in RTPCR.

Analysis of mRNA (converted to cDNA) was performed. The presence of amplification suggested positive *MYBL1*-trunc2 (**Figures 4A,B**). Four samples were positive for *MYBL1*-trunc2: nos. 50 (ganglioglioma), 58 (pilocytic astrocytoma), 60 (pleomorphic xanthoastrocytoma), and 52 (diffuse astrocytoma) (Table 4). Four samples were negative for *MYBL1*-trunc2: nos. 54 (pleomorphic xanthoastrocytoma), 62 (oligodendroglioma), 53 (pilomyxoid astrocytoma), and 61 (pleomorphic xanthoastrocytoma).

### RNA Sequencing

RNA sequencing results for *BRAF* showed no genetic alteration in 6 samples examined, probably because of low coverage in the area. On Sanger sequencing, 2 patients had the *BRAF* V600E genetic alteration.

**Figure 5:**
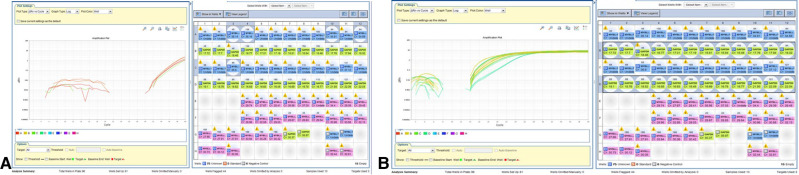
Positive MYBL1-trunc2. (**A**) Detection of MYBL-1 trunc 2 positive samples are shown:50(45), 58(138), 60(55), 52(84). Negative 4 samples: 54(158) PXA, 168 ODG, 53 (110) PMA, and 121 PXA. (**B**) Expression of normalized GAPDH gene in grade II gliomas samples.

RNA sequencing analysis of *CDKN2A* revealed one genetic alteration with high coverage quality, rs11515, in 5 of 6 patients. RT-PCR was performed on 9 grade II LGG samples. Deletions were detected in one of them (no. 54) in both exon 1 and exon 2 of *CDKN2A* (**Figure 5**).

RNA sequencing of *IDH1* and I*DH2* revealed no genetic alteration in *IDH1* and 13 potential single nucleotide polymorphisms (SNPs) in *IDH2* (*n* = 6). Eight of the SNPs were known intron variants with no pathogenic effect, 3 were unknown intron variants and were found to be heterozygous, and 2 were homozygous unknown intron variants and are under investigation.

## Discussion

In the present study, we used multiple newly implemented techniques to investigate some of the genetic alterations in IDH1, IDH2, CDKN2A, MYB and MYBL1 in pediatric low-grade gliomas. All these genes are relevant to the pathogenesis of LGGs, particularly the less studied and less common grade II gliomas. Nowadays, with the accumulation of data, the preferred method should be NGS (11, 19, 20).

In the present study, we showed that none of the LGGs had a genetic alteration in *IDH1/2*. This finding is in line with reports in the literature that *IDH1/2* genetic alterations appear almost exclusively in adult gliomas (10, 21). In adults with grade II-III gliomas, lack of an *IDH* genetic alteration indicates higher risk of transformation to a higher-grade tumor and poorer prognosis (22). Additionally, the grade II gliomas most often associated with *BRAF* genetic alterations were pleomorphic xanthoastrocytomas, similar to the findings of Myung et al. (23) Most samples with *MYB- PCDHGA1* translocation and *MYBL1* genetic rearrangement did not have a BRAF genetic alteration (9). The rate of BRAF genetic alteration in the grade II samples (3/9, 33%) was significantly higher than the reported rate for grade I pilocytic astrocytoma (13). We found MYB- translocations with exon 2 or 3 of PCDHGA1 5/10 and 4/10 grade II gliomas respectively, and *MYBL1* rearrangements in 6/9 of the samples examined. In grade II gliomas, the *MYB* translocations (with exon 2 of *PCDHGA1* in 5 samples and exon 3 of *PCDHGA1* in 4 samples) and the *MYBL1* rearrangement (in 6 samples) were technically difficult to prove and validate, which could serve as an indication for differentiating grade I from grade II-III gliomas, which have a potentially more aggressive outcome. These data are higher than reported elsewhere, and further studies and analytical methods are required.

Our detection of different combinations of *BRAF* genetic alterations, *MYB- PCDHGA1* translocations, and *MYBL1* rearrangement in the same samples contradicts the hypothesis of Zhang et al. (21) that *BRAF* and *MYB I* genetic alterations are mutually exclusive. The limited amount of sample material that was available for the study prevented us from confirming or repeating the results. Nevertheless, given that we used several different methods (PCR, RT-PCR, RACE, RNA sequencing), we assume the findings are correct.

Although Ramiksson et al. (9) showed the same results when analyzing DNA and cDNA (mRNA), we were unable to find a complete match. The DNA results are most likely more reliable, as mRNA is sensitive and minimal material was available. Samples 45 (ganglioglioma) and 84 (diffuse astrocytoma) were positive by all methods, but samples 158 (pleomorphic xanthoastrocytoma), 168 (oligodendroglioma), and 110 (pilomyxoid astrocytoma) were positive only on DNA analysis. (Some findings were confirmed in gel.) We cannot explain the positive cDNA and negative DNA results of sample 55.

In this study, we were pioneers in the implementation of new molecular methods to the analysis of data of our cohort. Further efforts must be taken to keep involved in the state-of-the art molecular methods, understand and use novel methods to detect translocations, genetic rearrangements, and abnormal levels of expression of several candidate genes in pediatric LGGs (23, 24). Our results are in line with the recent leading publications (6, 9, 10, 24) although we need to further explain the relative high occurrence of genetic alterations detected here in MYBL and MYB. At present, chemotherapy and radiotherapy are the mainstay of treatment for unresectable or progressive disease. It is clear that genetic abnormalities and activating genetic alterations are significant events in tumorigenesis in pediatric LGG and further exploration of the molecular pathways underlying their development will pave the way for the formulation of targeted treatments. *BRAF* inhibitors are already in use in the treatment of BRAF mutated high and low grade gliomas LGGs (25, 26). cMYC inhibitor may hold promise for the management of tumors caused by *MYB* and *MYBL1* translocations, Downstream MEK inhibitors are already in phase 2 in clinical trials for children with BRAF fused gliomas and phase 1 studies with FGFR1 inhibitors have started recently.

## Methods

### Clinical Specimens

Archival samples of pediatric LGGs (WHO grades I–II) were obtained from the Department of Pathology, Rabin Medical Center. Fresh tissues were also collected and analyzed after informed consent was obtained from the parents. Inclusion criteria were patient age <18 years and sufficient paraffin-embedded or frozen tissue for analysis. Tumor classification and cellularity were reviewed by a neuropathologist. Demographic and clinical data were collected from the medical files. The study was approved by the national and institutional review boards.

### DNA Extraction

Paraffin-embedded tissues or frozen tissues were used for DNA isolation. In brief, 10 µm sections were stained with hematoxylin-eosin, and the slides were reviewed by a pathologist. The areas with an estimated content of more than 75% tumor cells were then separated by microdissection (no. 11 surgical blade) from 5 consecutive 10-µm unstained paraffin-embedded sections of each block. The tissues were deparaffinized and incubated overnight in 1% sodium dodecyl sulfate and proteinase K 0.5 mg/mL. DNA was purified by phenol chloroform extraction and ethanol precipitation and dissolved in 50 µL of distilled water, as described previously (13). All fresh tissues removed at surgery were taken from areas immediately adjacent to the tumor that was submitted to pathology.

### Extraction of mRNA and Complementary DNA Preparation

Tumor tissue was snap-frozen in liquid nitrogen. Total RNA was isolated using TRIzol reagent (Invitrogen, Life Technologies, Carlsbad, CA) according to the manufacturer’s protocol and then reverse transcribed into complementary DNA (cDNA) using random hexamers (Amersham Biosciences, Buckinghamshire, United Kingdom) and Moloney murine leukemia virus reverse transcriptase (Promega, Madison, WI).

**IDH1 and IDH2 analysis** using specific designed primers for Sanger analysis.

### CDKN2A Deletion Analysis by RT-PCR

RT-PCR was used to check deletions in *CDKN2A.* The ratio between the *CDKN2A* gene Ct and the reference gene RNase Ct was calculated. A ratio of <0.5 was considered a deletion. The primers for exons 1 and 2 of *CDKN2A* and the reference gene RNase are shown in Table 5 (24).

**Table 5:** List of primers for CDKN2A.

CDKN2A exon 1	5′-CAACGCACCGAATAGTTACG-3′	5′-CTGCAAACTTCGTCCTCCAG-3′
CDKN2A exon 2	5′-ACCAGAGGCAGTAACCATGC-3′	5′-TGGAAGCTCTCAGGGTACAAA-3′
RNase	5′-GGGAGATGCGGAAGAATGT-3′	5′-CCTCCAGTCAGCCACAGAA-3′

### MYB and MYBL1 Genetic Alteration Analysis using RACE

Oncogenic genetic alterations in *MYB and MYBL1* were analyzed with rapid amplification of cDNA ends (RACE) using specific primers designed for the RACE reaction. The method entails initiation of first strand cDNA synthesis at the poly(A) tail of mRNA using the adapter primer and then destroying the original mRNA template with RNase. Specific cDNA is amplified by PCR using a gene-specific primer, which anneals in a region of known exon sequences, and an adapter primer that targets the 5´ terminus. The primers are listed in Table 6.

**Table 6:** List of primers for RACE.

#	chr	Orientation	Location	Primer	
1	6	Sense-axon initiation	MYB-Exon 8	ACATGCAGCTACC	Primer for initiation of the exon
2	6	Sense-nested	MYB-Exon8	CACCAGACCTCATGGAGACA	Short distance from previous primer
3	–	Antisense- Poly A	3′	CAUCAUCAUCAUGACCGTTCAGCTGGATATTAC	Universal primer for POLY A

### MYB Translocation and MYBL1 Rearrangement Analysis by PCR

To identify *MYB* translocation and *MYBL1* rearrangement, RNA was extracted from the tissue and converted to cDNA, as described (13). Triplicates of cDNA from LGG samples were analyzed. The mastermix contained 1 µg (1 µL), forward primer 0.5 µL (10 µM), reverse primers 0.5 µL (10 µM), Syber Fast 5 µL and H_2_O 3 µL. The total reaction volume (10 mL) was loaded into a 96-well plate and run on StepOne System software, v 2.2 (Applied Biosystems, Foster City, CA, USA) in real time. The primers are listed in **Table 2**. The Ct levels of product expression were calculated.

### MYB and BRAF Comparative Analysis

To investigate whether *MYB* and *BRAF* genetic alterations are mutually exclusive, we analyzed *MYB* and *BRAF* rearrangements in the same samples.

### BRAF Point Mutation and MYBL1 Rearrangement by PCR

After DNA extraction, each PCR amplification was performed in a 50 µL reaction volume containing 150 ng of sample DNA as a template using specific primers for the relevant genes. PCR parameters were as follows: denaturation for 5 min at 95°C; 35 cycles of 1 min at 95°C; and annealing for 1 min at 56–60°C and for 1 min at 72°C with Taq polymerase. The PCR product was amplified on 2% agarose gel and visualized with ethidium bromide staining.

### Validation

Direct sequencing of the PCR products was performed with reagents and an analyzer (Big Dye Terminator Cycle Sequencing and ABI PRISM 3700 DNA Analyzer; Applied Biosystems).

### RNA Sequencing

For RNA sequencing, we used 5 samples of grade II LGGs (2 pilomyxoid astrocytoma, 2 diffuse astrocytoma, 1 pleomorphic xanthoastrocytoma) and one sample of grade I LGG (pilocytic astrocytoma).

## Data Availability

The original contributions presented in the study are included in the article/supplementary material, further inquiries can be directed to the corresponding author/s.

## References

[1] Tatevossian RG Lawson ARJ Forshew T Hindley GFL Ellison DW Sheer D. MAPK pathway activation and the origins of pediatric low-grade astrocytomas. J Cell Physiol. (2010) 222:509–14. 10.1002/jcp.21978 19937730

[2] Gnekow AK Kortmann RD Pietsch T Emser A. Low grade chiasmatic-hypothalamic glioma - carboplatin and vincristin chemotherapy effectively defers radiotherapy within a comprehensive treatment strategy. Klin Pädiatr. (2004) 216:331–42. 10.1055/s-2004-832355 15565548

[3] Ohgaki H Kleihues P. Population-based studies on incidence, survival rates, and genetic alterations in astrocytic and oligodendroglial gliomas. J Neuropathol Exp Neurol. (2005) 64:479–89. 10.1093/jnen/64.6.479 15977639

[4] Fisher PG Tihan T Goldthwaite PT Wharam MD Carson BS Weingart JD Outcome analysis of childhood low-grade astrocytomas. Pediatr Blood Cancer. (2008) 51:245–50. 10.1002/pbc.21563 18386785

[5] Lyustikman Y Momota H Pao W Holland EC. Constitutive activation of Raf-1 induces glioma formation in mice. Neoplasia. (2008) 10:501–10. 10.1593/neo.08206 18472967 PMC2373912

[6] Ryall S Zapotocky M Fukuoka K Nobre L Guerreiro Stucklin A Bennett J Integrated molecular and clinical analysis of 1,000 pediatric low-grade gliomas. Cancer Cell. (2020) 37:569–83.e5. 10.1016/j.ccell.2020.03.011 32289278 PMC7169997

[7] Pfister S Janzarik WG Remke M Ernst A Werft W Becker N BRAF gene duplication constitutes a mechanism of MAPK pathway activation in low-grade astrocytomas. J Clin Invest. (2008) 118:1739–49. 10.1172/JCI33656 18398503 PMC2289793

[8] Eisenhardt AE Olbrich H Röring M Janzarik W Anh TN Cin H Functional characterization of a BRAF insertion mutant associated with pilocytic astrocytoma. Int J Cancer. (2011) 129:2297–303. 10.1002/ijc.25893 21190184

[9] Ramkissoon LA Horowitz PM Craig JM Ramkissoon SH Rich BE Schumacher SE Genomic analysis of diffuse pediatric low-grade gliomas identifies recurrent oncogenic truncating rearrangements in the transcription factor MYBL1. Proc Natl Acad Sci USA. (2013) 110:8188–93. 10.1073/pnas.1300252110 23633565 PMC3657784

[10] Ryall S Tabori U Hawkins C. Pediatric low-grade glioma in the era of molecular diagnostics. Acta Neuropathol Comm. (2020) 8. 10.1186/s40478-020-00902-z PMC7066826 32164789

[11] Jones DTW Kieran MW Bouffet E Alexandrescu S Bandopadhayay P Bornhorst M Pediatric low-grade gliomas: next biologically driven steps. Neuro-Oncology. (2018) 20:160–73. 10.1093/neuonc/nox141 29016845 PMC5786244

[12] Miklja Z Pasternak A Stallard S Nicolaides T Kline-Nunnally C Cole B Molecular profiling and targeted therapy in pediatric gliomas: review and consensus recommendations. Neuro-Oncology. (2019) 21:968–80. 10.1093/neuonc/noz022 30805642 PMC6682212

[13] Laviv Y Toledano H Michowiz S Dratviman-Storobinsky O Turm Y Fichman-Horn S BRAF, GNAQ, and GNA11 mutations and copy number in pediatric low-grade glioma. FEBS Open Bio. (2012) 2:129–34. 10.1016/j.fob.2012.05.004 23650591 PMC3642131

[14] Broniscer A Baker SJ West AN Fraser MM Proko E Kocak M Clinical and molecular characteristics of malignant transformation of low-grade glioma in children. J Clin Oncol. (2007) 25:682–9. 10.1200/JCO.2006.06.8213 17308273

[15] Dougherty MJ Santi M Brose MS Ma C Resnick AC Sievert AJ Activating mutations in BRAF characterize a spectrum of pediatric low-grade gliomas. Neuro-Oncology. (2010) 12:621–30. 10.1093/neuonc/noq007 20156809 PMC2940652

[16] Pollack IF Hamilton RL James CD Finkelstein SD Burnham J Yates AJ Rarity of PTEN deletions and EGFR amplification in malignant gliomas of childhood: results from the Children’s Cancer Group 945 cohort. J Neurosurg. (2006) 105(5 Suppl):418–24. 10.3171/ped.2006.105.5.418 17328268

[17] Tatevossian RG Tang B Dalton J Forshew T Lawson AR Ma J MYB upregulation and genetic aberrations in a subset of pediatric low-grade gliomas. Acta Neuropathol. (2010) 120:731–43. 10.1007/s00401-010-0763-1 21046410 PMC3066475

[18] Von Deimling A Korshunov A Hartmann C. The next generation of glioma biomarkers: MGMT methylation, BRAF fusions and IDH1 mutations. Brain Pathol. (2011) 21:74–87. 10.1111/j.1750-3639.2010.00454.x 21129061 PMC8094257

[19] Rubin JB Finlay JL. Pediatric low-grade gliomas: a brave new world. Neuro-Oncology. (2018) 20:149–50. 10.1093/neuonc/nox221 29365202 PMC5777489

[20] Packer RJ Pfster S Bouffet E Avery R Bandopadhayay P Bornhorst M Pediatric low-grade gliomas: implications of the biologic era. Neuro-Oncology. (2017) 19:750–61. 10.1093/neuonc/now209 27683733 PMC5464436

[21] Zhang J Wu G Miller CP Tatevossian RG Dalton JD Tang B Whole-genome sequencing identifies genetic alterations in pediatric low-grade gliomas. Nat Genet. (2013) 45:602–12. 10.1038/ng.2611 23583981 PMC3727232

[22] Yan H Parsons DW Jin G McLendon R Rasheed BA Yuan W IDH1 and IDH2 mutations in gliomas. N Engl J Med. (2009) 360:765–73. 10.1056/NEJMoa0808710 19228619 PMC2820383

[23] Myung JK Cho H Park CK Kim SK Lee SH Park SH. Analysis of the BRAF(V600E) mutation in central nervous system tumors. Translat Oncol. (2012) 5:430–6. 10.1593/tlo.12328 PMC3542839 23323158

[24] Bieńkowski M Piaskowski S Stoczyńska-Fidelus E Szybka M Banaszczyk M Witusik-Perkowska M Screening for EGFR amplifications with a novel method and their significance for the outcome of glioblastoma patients. PLoS One. (2013) 8:e65444. 10.1371/journal.pone.0065444 23762372 PMC3675194

[25] Raabe E Kieran MW Cohen KJ. New strategies in pediatric gliomas: molecular advances in pediatric low-grade gliomas as a model. Clin Cancer Res. (2013) 19:4553–8. 10.1158/1078-0432.CCR-13-0662 23881924 PMC4696061

[26] Hummel TR Chow LM Fouladi M Franz D. Pharmacotherapeutic management of pediatric gliomas : current and upcoming strategies. Paediatr Drugs. (2013) 15:29–42. 10.1007/s40272-012-0002-4 23329387

